# A Glimpse in the Future of Malignant Mesothelioma Treatment

**DOI:** 10.3389/fphar.2021.809337

**Published:** 2021-12-15

**Authors:** Gaetano Pezzicoli, Mimma Rizzo, Martina Perrone, Silvia Minei, Luciano Mutti, Camillo Porta

**Affiliations:** ^1^ Department of Biomedical Sciences and Human Oncology, University of Bari Aldo Moro, Bari, Italy; ^2^ A.O.U. Consorziale Policlinico di Bari, Bari, Italy; ^3^ Italian Group for Research and Therapy for Mesothelioma (GIMe), Voghera, Italy

**Keywords:** mesothelioma, immune checkpoint inhibitors, antiangiogenics, mesothelin, microRNA, oncolytic viruses, CAR-T cells

## Abstract

Malignant mesothelioma (MMe) is a rare neoplasm with few therapeutic options available. The landscape of effective therapy for this disease remained unchanged in the last two decades. Recently, however, the introduction of Immune Checkpoint Inhibitors (ICIs) led to small, but nevertheless, promising improvements. However, many efforts are still needed to radically improve the prognosis of MMe. In this review, we analyze all those therapeutic strategies for MMe that are still in a preclinical or early clinical phase of development. In particular, we focus on novel antiangiogenic drugs and their possible combination with immunotherapy. Furthermore, we describe also more complex strategies such as microRNA-loaded vectors, oncolytic viruses, and engineered lymphocytes.

## Introduction

Malignant Mesothelioma (MMe) is commonly considered a rare cancer. However, it is estimated to affect 40.000 people worldwide, with its incidence growing decade after decade (Odgerel et al., 2017; Keshava et al., 2019). MMe onset is heavily correlated with professional exposure to asbestos, and its low survival rate makes it the deadliest occupational disease ever known. A decrease in the number of new diagnoses in western industrialized countries is expected in the next few decades, due to governments programs of asbestos banishment. On the other hand, a dramatic rise is foreshadowed in all those second and third-world countries in which no action against this cancerogenic mineral has been taken yet.

MMe first clinical manifestation is often dyspnea due to pleural effusion. Less common presentations include chest pain or systemic symptoms such as fever, weight loss, or night sweats. The onset of these signs in a patient with a known history of professional exposure to asbestos must be seen as a red flag. In this setting, a chest computed tomography (CT) scan should be performed. Whenever pleural effusion and pleural nodules are detected through imaging, the next step consists of surgical biopsy or CT-guided biopsy. Even if the cytologic diagnosis can be very effective and less invasive, tissue biopsy is still to be preferred since it allows the detection of peculiar architectural patterns that have a prognostic role. Histological workup will differentiate mainly between three forms of MMe: epithelioid, sarcomatoid, and biphasic, with sarcomatoid differentiation being a negative prognostic index (Nicholson et al., 2020).

Even if metastases are a rare phenomenon in MMe, this tumor has a very poor prognosis due to the respiratory function impairment that it causes. Very few chemotherapy agents have shown some efficacy against this disease, and no major improvements in its treatment occurred in the last two decades. Recently, however, something new emerged, leading to the introduction of a whole new class of drugs in the management of MMe: the immune checkpoint inhibitors (ICI).

## Current and Upcoming Standard of Care

### Surgery

Pleural decortication and extrapleural pneumonectomy are the two major surgical techniques involved in the treatment of MMe. Unfortunately, no consensus exists about the role of surgery in the management of MMe, since there is a lack of prospective studies. The MARS trial is one of the few studies of this type and it concludes that there is no significant survival advantage in patients undergoing extrapleural pneumonectomy, followed by palliative chemotherapy, compared with patients receiving palliative chemotherapy alone (HR = 1.90, 95% CI = 0.92–3.93; *p* = 0.082) (Treasure et al., 2011). The precise role of pleural decortication is being investigated within the MARS-2 trial, which is still recruiting and its completion is estimated in late 2022 (NCT02040272).

What emerges from retrospective analyses is the importance of patient selection. Factors such as age, comorbidities, and performance status are important guidance in this choice. Also, histology plays an important role: many experts point out that sarcomatoid MMe should never receive surgical treatment, while the debate is still open for biphasic MMe (Ricciardi et al., 2018).

Neoadjuvant treatments have been the object of active investigation, yielding mixed results. As a whole, neoadjuvant chemotherapy granted no survival advantage (Voigt et al., 2020). Neoadjuvant radiotherapy was recently evaluated in a phase II trial (SMART), that showed only the feasibility of this technique (Cho et al., 2021).

However, even though there is a certain interest in the surgery of MMe, it should be noted that the vast majority of patients are be suitable for surgical intervention, and will receive a palliative systemic therapy only.

### Palliative Chemotherapy

Patients unsuitable for surgery, usually receive first-line chemotherapy. The combination of Pemetrexed and Cisplatin represents the backbone of the treatment in this setting since a phase III trial demonstrated a progression-free survival (PFS) improvement for the combination versus cisplatin alone (5.7 vs. 3.9 months, *p* = 0.001), back in 2003 (Vogelzang et al., 2003). Carboplatin can be used in patients unfit for Cisplatin (Ceresoli et al., 2006). There is currently no evidence supporting maintenance therapy for patients achieving disease control, with many studies demonstrating no benefit from the prosecution of Pemetrexed, the latest being the CALGB 30901 trial (Dudek et al., 2020). The addition of antiangiogenics to chemotherapy has been considered since MMe is well known for its high VEGFR expression (Aoe et al., 2006). The MAPS trial observed a slight advantage in overall survival (OS) for patients receiving Pemetrexed, Cisplatin, and Bevacizumab compared with patients receiving chemotherapy alone (18.8 vs. 16.1 months, HR 0.77, *p* = 0.0167). However, this improvement comes at the cost of a higher rate of grade 3–4 adverse events (Zalcman et al., 2016). As a whole, Bevacizumab is not FDA approved in this setting.

Only a few options exist in the common clinical practice for patients with progressive disease after first-line therapy. For patients experiencing progression more than 6 months from the first-line treatment, rechallenge with pemetrexed and a platinum compound is advised. Bearz et al. demonstrated that in this setting, the rechallenge strategy allows a disease control rate (DCR) of 66%, with a PFS of 5.1 months and an OS of 13.6 months (Bearz et al., 2012). However, patients suitable for rechallenge represent a minority. For patients experiencing early progression, monotherapy with Gemcitabine or Vinorelbine seems to be one of the few strategies offering a minimal survival improvement (Zucali et al., 2014). Recently, the RAMES trial showed a possible role for antiangiogenic drugs in this setting. The combination of Gemcitabine and Ramucirumab (an anti-VEGR2 monoclonal antibody) was compared with Gemcitabine alone in patients beyond the first line of treatment, obtaining a significant OS improvement (13.8 vs. 7.5 months, HR 0.71, 70% CI 0.59–0.85; *p* = 0.028) (Pinto et al., 2021), with an acceptable safety profile. However, this study has been criticized especially for the positive selection of the patients enrolled (Porta et al., 2021).

### Immune Checkpoint Inhibitors

The introduction of ICIs can be considered a turning point in the management of MMe. In fact, after many studies showing a potential role for this treatment in the second and third lines, (Fennell et al., 2018; Popat et al., 2020) the Checkmate-743 trial demonstrated an important clinical improvement with an ICI in the first line setting (Baas et al., 2021). Specifically, this study randomized 605 patients with advanced MMe to receive either Nivolumab (an anti-PD-L1 monoclonal antibody) plus Ipilimumab (an anti-CTLA4 monoclonal antibody), or platinum-based chemotherapy. The OS was 18.1 vs. 14.1 months (HR 0.74, *p* = 0.002) and the frequency of the adverse events was comparable between the two groups. Subgroup analyses showed benefits for the immune combo in nearly every considered subgroup, with only a minor (but nevertheless significant) improvement for patients with PD-L1 negative tumors, as well as in elderly patients. A finding of high interest is the heightened efficacy of the immune combo compared with chemotherapy in patients with non-epithelioid histologies (OS = 18.1 vs. 8.8 months, HR = 0.46). On the other hand, it should be noted that this trial has some limitations, since it did not enroll patients with an ECOG Performance Status of 2, which are not a rare among the population of MMe patients undergoing active treatments. In the meantime, the study population was still so fragile that small changes in the randomization could have lead to different statistical conclusions.

Other trials are trying to expand the role of ICIs in MMe. The DREAM phase II trial showed a 6.7 months PFS and a 20 months OS in naïve MMe patients treated with Durvalumab (an anti-PD-L1 monoclonal antibody) plus Pemetrexed and Cisplatin (Nowak et al., 2020). This could be a reasonable therapeutic option for patients with epithelioid histology. Finally, the phase III BEAT-meso trial is currently enrolling naïve patients with MMe to evaluate the combination of Pemetrexed, Carboplatin, Atezolizumab, and Bevacizumab (NCT03762018). Results are expected in early 2024.

As emerges from this brief overview, in the last few years many new therapeutic targets approached the common clinical management of MMe. After more than 15 years without significant changes, the standard of care has been ultimately modified by these novel findings. On the other hand, it should be considered that MMe still remains a disease with few overall therapeutic options, even including the most recent breakthroughs. Therefore, the research for new targets and, consequentially, new therapeutic strategies, will be an important aim for the next decade.

## Novel Agents and Combinations

The results of the studies analyzed in this section are summarized in Table 1.

**TABLE 1:** Clinical trials involving novel therapeutic strategies for MPM.

Strategy exploited	Study population	Study design	Outcome	References
Novel cytotoxic agents	30 patients with MPM, that had progression after a first-line platinum-based therapy	A phase II trial investigating the efficacy of **BNC105P** (tubulin-targeting agent)	ORR 3%, DCR 43%, mOS 8.2 months, no grade 3–4 AE	Nowak et al. (2013)
	42 patients with MPM, that had progression after first-line platinum-based therapy or immunotherapy	SAKK 16/17: phase II trial investigating the efficacy of **Lubrinectidin** (DNA transcription inhibitor)	ORR 4%, DCR 52%, mOS 11.1 months, mPFS 4.1 months	Metaxas et al. (2020)
Arginine deiminase	386 treatment-naïve patients with MPM (ongoing enrollment)	ATOMIC-meso: phase II/III trial investigating the efficacy of **ADI-PEG20** (pegylated arginine deiminase) in combination with pemetrexed and cisplatin	Not yet published	NCT02709512
Tyrosin-Kinase inhibitors	24 patients with MPM, that had progression after first or second-line chemotherapy	A phase II trial investigating the efficacy of **AZD4547** (FGFR1-3 inhibitor)	Discontinuation due to low 6-months-PFS (12%)	Lam et al. (2020)
	62 patients with pretreated advanced neoplasms (including a cohort of 29 MPM)	A phase I pharmacokinetic and pharmacodynamic study of **GSK2256098** (focal adhesion kinase inhibitor)	ORR 10%, mPFS 3 months	Soria et al. (2016)
Antiangiogenics	54 patients with MPM, that had progression after a first-line platinum-based therapy	SWOG S0509: a phase II trial investigating the efficacy of **Cediranib** (pan-VEGFR inhibitor)	ORR 9%, DCR 42%, mOS 9.5 months, mPFS 2.6 months. 91% of patients required dose reduction	Garland et al. (2011)
	51 patients with MPM, that had progression after a first-line platinum-based therapy	A phase II trial investigating the efficacy of **Cediranib** (pan-VEGFR inhibitor)	ORR 10%, DCR 67%, mOS 4.4 months, mPFS 1.9 months. 87% of patients reported grade 3/4 AE	Campbell et al. (2012)
	92 treatment-naïve patients with MPM	SWOG S0905: a phase II comparison of **Cediranib** (pan-VEGFR inhibitor) + pemetrexed and cisplatin vs pemetrexed and cisplatin	Increased DCR (50 vs 20%) and mPFS (7.2 vs. 5.6 months) in the cediranib arm. No differences in mOS. More AE in the ceridanib arm	Tsao et al. (2019)
Combination of ICI and antiangiogenics	18 patients with solid neoplasms, including MPM, that had progression after first-line therapy (ongoing enrollment)	PEMBIB: a phase I trial investigating safety and activity of **Pembrolizumab** and **Nindetanib**	Not yet published	NCT02856425
	400 treatment-naïve patients with MPM (ongoing enrollment)	BEAT-meso: a phase III trial of **Pemetrexed-Carboplatin-Bevacizumab-Atezolizumab** vs Pemetrexed-Carboplatin-Bevacizumab	Not yet published	NCT03762018
	20 patients with malignant peritoneal mesothelioma, that had progression after first-line platinum-based therapy	A phase II trial investigating the efficacy of **Atezolizumab plus Bevacizumab**	ORR 40%, 1-year-PFS 61%, 1-year-OS 85%	Raghav et al. (2021)
DNA reparation impairment	74 patients with MPM, that had progression after first-line platinum-based therapy	A phase II trial investigating the efficacy of **Tamezostat** (EZH2 inhibitor)	ORR 3%, 12-weeks-DCR 51%, 24-weeks-DCR 25%	Marjorie et al. (2020)
microRNA	26 patients with MPM, that had progression after first-line platinum-based therapy	A phase I trial investigating safety and activity of **TargomiRs** (miR16-loaded non-living minicells)	ORR 5%, DCR 73%, mOS 6.8 months	van Zandwijk et al. (2017)
HDAC inhibitors	661 patients with MPM, that had progression after first or second-line chemotherapy	VANTAGE-014: a phase III trial of **Vorinostat** vs placebo	No significant improvement in OS with Vorinostat (mOS 7.5 vs. 6.7 months)	Krug et al. (2015)
Novel immunotherapies	148 patients with pretreated advanced neoplasms (including MPM)	A phase I Dose-Escalation trial of **Anetumab Ravtansine** (Anti-Mesothelin Antibody-Drug Conjugate)	ORR 8%, DCR 52%, best responses in tumor with high mesothelin expression	Hassan et al. (2020)
	248 patients with MPM, that had progression after first-line platinum-based therapy	A phase II trial investigating the efficacy of **Anetumab Ravtansine** (Anti-Mesothelin Antibody-Drug Conjugate)	Not yet published	NCT02610140
	35 treatment-naïve patients with MPM	A phase I trial investigating safety and activity of **CRS-207** (Listeria monocytogenes Expressing Mesothelin) with Chemotherapy	ORR 57%, DCR 86%, mOS 14.7 months, mPFS 7.5 months	Hassan et al. (2019)
	27 patients with MPM, that had progression after first-line platinum-based therapy	A phase I trial investigating safety and activity of intrapleural **CAR-Tmeso plus Pembrolizumab**	mOS 23.1 months, 1-year-OS 83%	Adusumili et al. (2021)
Oncolytic viruses	13 patients with MPM, who received a maximum of one line of treatment	A phase I/II trial investigating the efficacy of **HSV1716** (oncolytic Herpesvirus)	ORR 0%, DCR 50%	Danson et al. (2020)

Abbreviations: mOS, median overall survival; mPFS, median progression-free survival; ORR, objective response rate; DCR, disease control rate; AE, adverse event. Bold values are the experimental drug(s) evaluated in each study.

### Novel Cytotoxic Chemotherapy Agents

Despite the above recent improvements, the search for novel chemotherapeutic agents continues. Among the many compounds tested in MMe, BNC105P is surely one of the most interesting.

BNC105P is a tubulin targeting agent that disrupts the vascular architecture in solid tumors. It has been demonstrated to enhance the effect of VEGFR-inhibitors and mTOR-inhibitors in renal and breast cancer (Inglis et al., 2014). This drug was also tested in MMe, witin a phase II trial enrolling patients that had progressed on first-line platinum-based therapy (Nowak et al., 2013). In spite of the absence of grade 3–4 adverse events, the reported ORR was just 3%, with a DCR of 43%, and a median OS of 8.2 months. As a whole, it is clear that BNC105P is not an option as a monotherapy. However, its good safety profile and the solid rationale behind its use in MMe are solid points that could justify further development of this agent within combination with other agents.

Another interesting compound is Lubrinectedin, an agent that is similar to Trabectedin and that showed good efficacy in small cell lung cancer that progressed beyond the first line of platinum-based therapy (Trigo et al., 2020). Lubrinectedin acts by binding specific DNA sites thus impeding the access of transcriptional machinery and by inhibiting the function of tumor-associated macrophages (Santamaría Nuñez et al., 2016). A preliminary *in vitro* study showed a high sensitivity of MMe cell lines to Lubrinectidin, regardless of the BAP1 status and histological subtype (Anobile et al., 2021). Specifically, the inhibition in tumor growth was caused by the stop of the cell cycle in the S-phase with the activation of the DNA damage response and consequent apoptotic cell death. Given these premises, Lubrinectedin was tested in the SAKK 17/16 phase II trial (Metaxas et al., 2020). MMe patients with at least one therapeutic line failure were given Lubrinectidin in monotherapy. Interestingly, immunotherapy was included among the possible previous therapeutic lines, making this trial one of the few available trials providing evidence of activity after ICIs. A median PFS of 4.1 months and a median OS of 11.1 months were reported. The safety profile was acceptable, with only a minor part of patients experiencing neutropenia and fatigue. These results are of great clinical interest since Lubrinectidin is among the few drugs that reported clinical efficacy after ICIs in MMe. Moreover, the possibilities for combination therapies including Lubrinectidin in this setting are completely open.

### Arginine Deiminase

Pegylated arginine deiminase is a novel therapeutic agent. In the Argininosuccinate synthetase 1 (ASS1)-deficient tumors, the enzyme Arginine Deiminase triggers an extracellular arginine depletion, inducing a pro-survival metabolic reprogramming that redirects glucose into the serine/folate pathway directing the carbons from glucose into pyrimidine biosynthesis, thus sensitizing cells to death by the pyrimidine antimetabolite, such as pemetrexed (Singh et al., 2019). The ASS1 deficit was identified in up to 75% of non-epithelioid MMe. Hence the rationale of the ATOMIC-meso phase II/III trial (NCT02709512) in which the ADI-PEG20 (pegylated recombinant Arginine Deiminase) is being tested in combination with Pemetrexed and Cisplatin, in naïve non-epithelioid MMe patients. In the phase II part of the study, patients are enrolled in an ASS1-agnostic fashion, with an option to restrict enrolment to ASS1-deficient in phase III. The control arm for phase III will be the platinum doublet. It is estimated that the study will be completed in late 2022.

### Tyrosine-Kinase Inhibitors

In the last two decades, TKIs changed the clinical management of a number of neoplasms, including kidney cancer and non-small cell lung cancer (NSCLC). However, the TKIs that worked for NSCLC showed little-to-no efficacy in MMe (Garland et al., 2007). Only recently, the expansion in the knowledge of MMe-specific drivers allowed the development of more promising TKIs.

Fibroblast Growth Factor Receptors (FGFR) 1–4 are often overexpressed in MMe, with FGFR3 and 4 being also predictors of shorter OS (Vlacic et al., 2019). A phase II clinical trial studied the efficacy of the FGFR 1–3 inhibitor AZD4547 in 24 advanced MMe patients that progressed after 1 or 2 lines of chemotherapy. However, the primary endpoint was not met, with a 6-months PFS of a mere 12%, which lead to study discontinuation (Lam et al., 2020).

Another potential therapeutic target could be the Focal Adhesion Kinase (FAK), which is known to be amplified in MMe and pancreatic cancer. Kanteti et al. demonstrated growth inhibition caused by FAK-inhibitors in an *in vitro* model of MMe (Kanteti et al., 2018). Based on this preclinical rationale, Soria et al. explored the role of the FAK-inhibitor GSK2256098 in a cohort of 62 patients with different solid tumors, within a phase Ib trial (Soria et al., 2016). The subgroup of patients with MMe (n = 29) had a mPFS of 12 weeks, and 3 of them experimented partial responses, a quite interesting finding taken into account that the patients enrolled were heavily pretreated. Moreover, a slightly better response was observed in MMe characterized with Merlin loss. The acceptable safety profile and the preliminary efficacy data, make FAK a target worth further experimentation.

Cyclin-dependent Kinases (CDK) are another interesting target. In the last decade, CDK 4/6 inhibitors have been game-changers in the treatment of metastatic breast cancer (Spring et al., 2019). Aliagas et al. observed that MMe patients with CDK4/6 overexpression experience a shorter survival. Therefore they tested the effects of the CDK 4/6 inhibitors Abemaciclib and Palbociclib on *in vitro* and *in vivo* preclinical models of MMe. Both the inhibitors showed the capability of blocking the cell cycle in the G1 phase and inducing senescence (Aliagas et al., 2021). Moreover, Abemaciclib was tested in a mouse model of MMe, achieving a significant inhibition of tumor growth, especially when combined with radiation therapy (Seiji et al., 2018). Another evidence supporting the use of CDK 4/6 inhibitors in MMe comes from an in silico analysis showing that the resistance to PD1-blockade in this tumor often comes from the deletion of CDKN2A (Jang et al., 2021). The authors of this analysis argued that this resistance mechanism can be overcome through the inhibition of CDK 4/6, and proceeded to demonstrate this hypothesis in an *in vivo* murine model. The combination of daily oral administration of CDK4/6 inhibitors (Abemaciclib or Palbociclib) and intraperitoneal anti-PD-1 treatment markedly suppressed tumor growth, compared with anti-PD-1 or CDK4/6 inhibitor alone. Therefore, there is a strong rationale for the use of CDK4/6 inhibitors, maybe in combination with ICIs. Presently, the currently recruiting MiST trial (NCT03654833) includes an arm of MMe patients selected by the p16/INK4A deficiency, who will receive Abemaciclib in monotherapy as a treatment. First results are expected in late 2021.

### Antiangiogenics in Monotherapy and in Combination

Antiangiogenic drugs may play an important role in the management of MMe. The pan-VEGFR inhibitor Cediranib has extensively been tested in this disease. Initially, it was tested as a second-line monotherapy treatment, to be used after progression to platinum-based chemotherapy. The SWOG S0509 phase II trial reported an mOS of 9.5 months, with an ORR of 9% (Garland et al., 2011). Due to the low efficacy and the high rate of dose reductions (91% of all the patients), the study was discontinued. Similar results were described in a phase II trial by Campbell et al., who reported an even lower mOS (4.4 months) (Campbell et al., 2012). More recently, however, Cediranib was evaluated in combination with cisplatin and pemetrexed in chemotherapy-naïve MMe patients. The phase II SWOG S0905 compared the combination of cediranib with chemotherapy, yielding a PFS benefit in favor of the combination arm (7.2 vs. 5.6 months, HR 0.71, *p* = 0.062), but no significant differences in OS (Tsao et al., 2019). As already seen in the RAMES trial, these studies seem to indicate a possible role for novel antiangiogenic therapies in MMe, especially when combined with other cytotoxic drugs.

A different approach could be the combination of ICIs and antiangiogenics. As observed in kidney cancer, the effects of ICIs and antiangiogenic drugs are synergistic (Rini et al., 2019).

The PEMBIB trial (NCT02856425) is a phase Ib trial currently enrolling patients with different solid malignancies to receive a combination of Pembrolizumab and Nindetanib. The study includes a cohort of patients with MMe and it is currently reporting a favorable safety profile for the combination (Andreea et al., 2018). However, the completion is expected in 2026. Another study exploiting the combination of antiangiogenics and immunotherapy is the previously mentioned BEAT-meso trial, which is evaluating the combination of Atezolizumab, Bevacizumab, and chemotherapy. Neither this study will be completed soon. Recently, interesting evidence comes from a small study that demonstrated the activity of the combination of Atezolizumab plus Bevacizumab in a cohort of patients with peritoneal mesothelioma that had failed one previous platinum-based therapy line (Raghav et al., 2021). An ORR of 40% was reported, with a median duration of response of 12.8 months, a 1-year-OS of 85%, and a 1-year-PFS of 65%. The response seemed to be independent of the PD-L1 status and the principal resistance mechanism observed was the epithelial-to-mesenchymal transition.

Whatsoever, more studies should be carried on in order to better understand the place for this combination in the future landscape of MMe therapies.

### Agents for MMe With Impairment in DNA Repair

A high DNA instability has been documented in MMe (Ivanov et al., 2009). Specifically, asbestos exposure seems to correlate with a decreased activity of the DNA reparation system based on the Poli-ADP-Ribose Polymerase 1 (PARP1). As a counterproof, MMe cells often display a high grade of DNA damage, despite PARP1 being overexpressed (Tomasetti et al., 2011). It should hereby be highlighted that many MMe cases show the loss of the BRCA-associated protein 1 (BAP1), which is a deubiquitinase strictly implied in the BRCA-dependent DNA reparation system. Therefore MMe can be pictured as a tumor with a strong impairment in the DNA reparation. A preliminary *in vitro* study demonstrated that PARP inhibitors do not exert a strong cytotoxic effect on MMe cell lines, di per se (Rathkey et al., 2020). This can be explained considering that, as described before, PARP activity should already be minimal in MMe. However, the same study proved that in BAP1-deficient MMe, the combination of Temozolomide and PARP inhibitors can limit tumor growth. This may be due to the DNA damage caused by Temozolomide, which cannot be repaired by these tumor cells that lack the necessary enzymes. Given the analyzed rationale and the preclinical results, the clinical experimentation of this combination could theoretically lead to promising outcomes.

There is, however, another drug that could bring novelty in the management of BAP1-deficient MPM. Tazemetostat is a specific inhibitor of the Enhancer of Zeste-Homolog 2 (EHZ2), a methyltransferase enzyme that plays a role in epigenetic regulation, by modulating the histones and by suppressing the transcription during cell division. The EHZ-203 phase II trial (NCT02860286) is evaluating the role of Tazemetostat in BAP1-deficient MMe, the rationale being the following: by inhibiting EHZ2, MMe will be unable to effectively suppress transcription, thus resulting in the production of aberrant proteins due to the DNA alteration linked to BAP1 loss (Marjorie et al., 2020). At the last update, the study reported a 12-weeks DCR of 47%, which is promising, considering that all the enrolled patients had failed at least one chemotherapy line. This trial both suggests a possible role for the combination of novel agents and presents an optimal paradigm for drug development in molecularly defined cohorts in mesothelioma.

### microRNA

**FIGURE 1:**
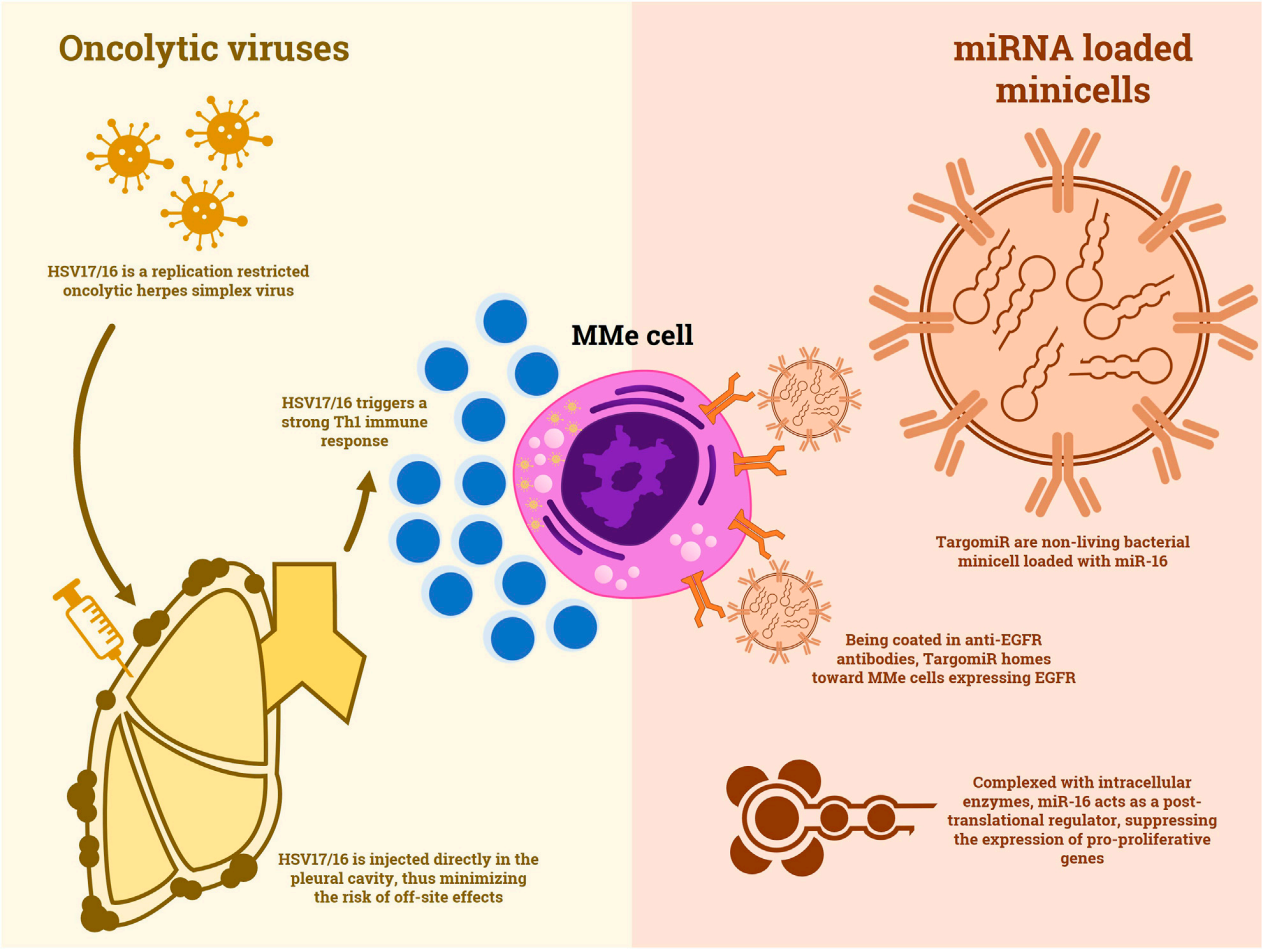
A comparison between the mechanisms of action of oncolytic viruses and non-living minicells loaded with miRNA.

Since their discovery, microRNAs have always been considered a potential anti-tumoral target. These non-coding small RNA molecules usually play a role in the regulation of gene expression at a post-transcriptional level, by combining with specific proteins, thus creating ribozymes that suppress coding mRNAs (Peng and Croce, 2016). miRNAs were often found to be altered in many different cancer types. In MMe, some oncosuppressive role has been reported for miR-15/16. Reid et al. described a decreased expression of miR-15/16 in MMe tumor specimens and cell lines (Reid et al., 2013). Moreover, they demonstrated that restoring miR-16 function in MMe xenograft-bearing mice leads to an inhibition of tumor growth correlated with the downregulation of Bcl-2 and CCND1. The most important detail of this *in vivo* experimentation is the device used to restore the microRNA expression. In fact, Reid et al. engineered nonliving bacterial minicells to carry miR-16 and made them target MMe cells by positioning bispecific anti-EGFR antibodies on their surface (Figure 1). This system would later become known as TargomiRs. A phase I trial of TargomiRs in patients with pretreated MMe (NCT02369198) was recently published (van Zandwijk et al., 2017). The safety profile of this treatment resulted to be acceptable, with principal adverse events being lymphopenia, hypophosphatemia and increased transaminasemia. Despite an ORR of 5%, the reported DCR was 73%, with an mOS of 6.5 months. Therefore, TargomiRs should be furtherly explored in the treatment of (NCT02369198), and combinations with immunotherapy or chemotherapy could be proposed.

### Histone Deacetylase Inhibitors

HDAC inhibitors are still struggling to find their place in the clinical management of cancer. The *in vitro* studies show good tumor inhibition capabilities derived from their epigenetic manipulation. However, many clinical trials have been concluded without convincing evidence of their efficacy as a monotherapy. The scenario is not different for MMe. Panobinostat was demonstrated to inhibit mesothelioma cell growth both *in vitro* and in mice models (Crisanti et al., 2009). In this study, the same effect was observed in immunocompetent and immunodeficient mice, therefore it was assumed that it did not depend upon the immune system clearance mechanisms. These data sounded promising for a clinical application of HDAC inhibitors. In this context, the VANTAGE-014 study was promoted (Krug et al., 2015). In this phase III trial, MMe patients who experienced progression after a first-line platinum-based treatment were randomized to receive Vorinostat or placebo as a second-line treatment. The median OS did not significantly differ between the two treatments (30.7 vs. 27.1 weeks *p* = 0.86). Even if this trial could be the tombstone on the use of HDAC inhibitors in MMe, a recent preclinical study rekindled the interest in this class of compounds. In an *in vitro* study, Bensaid et al. demonstrated that the combination of novel HDAC inhibitors and the hypomethylating agent Decitabine can induce the expression of specific immunogenic antigens on the surface of MMe cells and that these antigens are able to elicit specific immune responses (Bensaid et al., 2018). This effect, however, comes along with an increased PD-L1 expression. Therefore, this study foreshadows the possibility that a combination of HDAC inhibitors and ICIs could lead to increased efficacy of immunotherapy in MMe.

### Novel Immunotherapies

**FIGURE 2:**
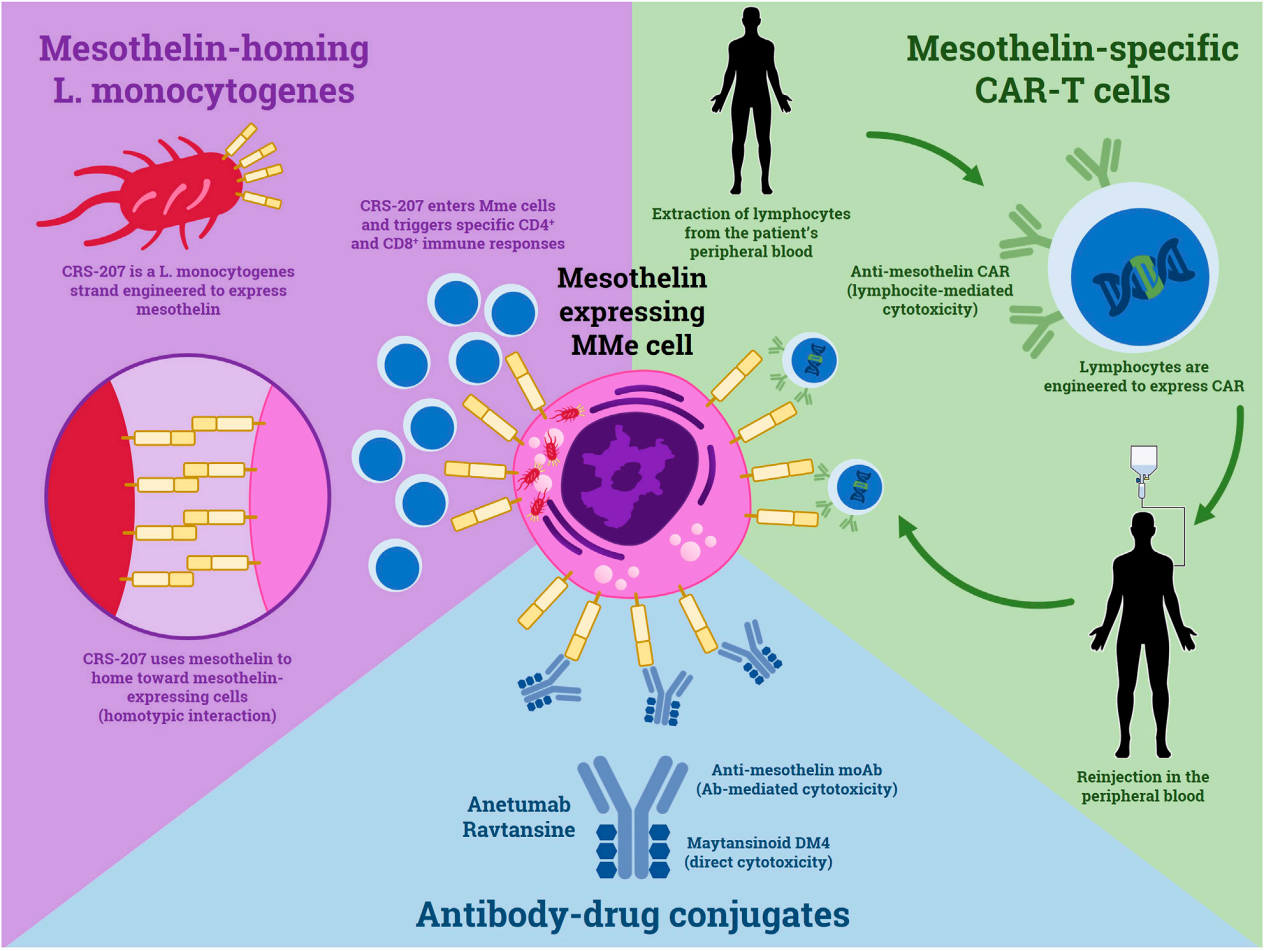
Mechanisms of the novel immunotherapy strategies which use mesothelin as a target.

The idea of using the immune system to target cancer has longly fascinated oncologists. In the last decade, the great success of ICIs demonstrated that this strategy could change the clinical practice for many different kinds of tumors. However, what has been done with ICIs is a mere scratch on the surface of what immunotherapy could produce. In fact, ICIs act by removing the natural boundaries of immune response thus allowing the immune system to overcome cancer immunoediting. However, more complex therapies that are currently under development, could take this concept to the next level. The strategies exploited in this chapter are summarized in Figure 2.

With respect to MMe, the evolution of immunotherapy passes through the identification of a specific marker, possibly expressed on the surface of cancer cells. Mesothelin seems to fit this role. The function of this protein is not completely understood: it is well known its role as a heterotypic adhesion and its interaction with the CA-125 antigen (Bononi et al., 2015). Given its overexpression on MMe cells, mesothelin could be implied in contact-mediated survival. All these characteristics make it an ideal target.

The development of an anti-mesothelin antibody has longly been pursued. Many preclinical studies were carried on, with mixed results. However, very recently, the first anti-mesothelin antibody with a conjugated cytotoxic drug reached clinical experimentation. Anetumab Ravtansine is an anti-mesothelin antibody linked to an antimicrotubular agent (maytansinoid DM4) that was recently tested in a phase I dose-finding trial, demonstrating an acceptable safety profile (Hassan et al., 2020). The study included patients with many different mesothelin-expressing tumors, often heavily pretreated. Nevertheless, a disease control rate of 53% was reported. In particular, the MMe cohort was the one with the best results. The activity of this conjugate seems to be higher in those patients with a higher expression of mesothelin. Recently, a phase II trial was completed. In this study (NCT02610140), MMe patients who had progression after platinum-based chemotherapy were randomized to either Anetumab Ravtansine or Vinorelbine. However, the results are still undisclosed.

A less orthodox way of targeting mesothelin has been tried by Hassan et al. (2019). In a phase Ib trial, they utilized a non-virulent strand of the bacteria Listeria monocytogenes engineered to express mesothelin, namely CRS-207. Exploiting the homotypic interaction of mesothelin molecules, CRS-207 reaches the MMe sites and here it triggers mesothelin-specific CD4+ and CD8+ T-cell responses, as demonstrated in preclinical studies (Le et al., 2015). In this phase Ib trial, patients with treatment-naïve MMe received induction with CRS-207, followed by up to 6 cycles of Cisplatin-Pemetrexed chemotherapy. Out of 35 patients, 31 had disease control, with 1 complete response and 19 partial responses. The median PFS and OS were 7.5 and 14.7 months, respectively. The most interesting part of this study is the comparison of pre and post CRS-207 biopsies, which showed a reduction of immunosuppressive phenomena and a switch of the immune infiltrate toward an active anti-cancer response. More studies on this novel strategy could feature combinations with other agents, leading to an even more effective response.

There is, however, an even more advanced strategy to target MPM cells that represents the pinnacle of next-level immunotherapies: the Chimeric Antigen Receptor T cells (CAR-T). Essentially, CAR-T are T lymphocytes engineered to express a chimeric receptor that is specific for a selected antigen. They represented a major breakthrough in the treatment of hematologic neoplasms in recent years. As for the management of MPM, some clinical trials featured mesothelin-specific CAR-T. In 2014, Beatty et al. exposed the first two cases of patients treated with CAR-Tmeso, a specific clone of CAR-T cells engineered to transiently express the CAR, thus limiting the possible onset of immune-related toxicities (Beatty et al., 2015). The report claims that the therapy is safe, feasible and it causes the onset of specific immune responses in the patient. CAR-Tmeso were successively tested in a phase I trial enrolling pretreated patients with mesothelin-expressing tumors, including MMe, ovarian cancer, and pancreatic ductal adenocarcinoma (Haas et al., 2019). CAR-T cells were well tolerated and in the majority of patients, their DNA having been found in tumor biopsies. Moreover, this study demonstrated the importance of lymphodepletion before the infusion of CAR-T cells, since this strategy favored the expansion of the engineered lymphocytes. But it was in an even more recent study, that CAR-Tmeso showed their true efficacy. In the phase I trial by Adusumili et al., CAR-Tmeso were administered through intrapleural infusion in 25 MMe patients that already received at least one line of therapy (Adusumilli et al., 2021). After these infusions, patients received immunotherapy with Pembrolizumab. The median OS from the first CAR-T infusion was 23.8 months, with 83% of patients alive at 1 year. Two patients exhibited a complete response at the PET scan. This study is of great interest not only for the management of MMe but also for the whole field of immunotherapy in solid tumors. The use of ICIs could represent the turning point in the struggle to export CAR-T therapy to non-liquid tumors. If these results will be confirmed by wider trials, the only limitations for this strategy will be its cost and its complexity.

### Oncolytic Viruses

The use of viruses to attack tumor cells and trigger the host immune response has been purposed many times in the last 30 years, even if its realization proved to be difficult. Only recently, the first oncolytic viruses are reaching clinical practice. An example is T-VEC an oncolytic virus used in the treatment of advanced melanoma, that recently completed its phase III trial (Andtbacka et al., 2019).

One of the first reports of an oncolytic virus used against MMe is a 1997 preclinical study in which a replication restricted HSV-1 showed efficacy against *in vitro* MMe cell lines and it demonstrated to inhibit tumor growth in mice xenografts (Kucharczuk et al., 1997). More recently, other viruses were purposed for the same strategy. Li et al. reported the *in vitro* and *in vivo* efficacy of a measles virus engineered to carry the interferon-beta gene to enhance immune response and the sodium iodide symporter gene to track the virus diffusion through SPECT (Li et al., 2010). This virus was able to effectively induce apoptosis in tumor cells and trigger a specific immune response. Similar results in nude mice were obtained with a vaccinia oncolytic virus by Belin et al. (2013). Finally, the first clinical results were obtained by a replication restricted herpes virus, HSV1716, in a phase I/II trial (Danson et al., 2020). Thirteen patients with advanced MMe, 8 of which were treatment-naïve, received intrapleural injections of this virus (Figure 1). Despite the absence of objective responses, disease stabilization was achieved in 50% of patients and no major adverse events were reported. Moreover, active viral replication and Th1 cytokines were documented in the pleural fluid of the patients. This study opens up to the possibility of an oncolytic virus therapy in MMe, however, studies of combination strategies, maybe with ICIs, could be beneficial.

### Hypoxia

Like many other forms of cancer, MMe shows a metabolic rewiring finalized to maintain the production of energy and to sustain the anabolic pathways in a hostile microenvironment. This is possible through the activation of specific regulators such as the Hypoxia Inducible Factors 1α and 2α (HIF-1α and HIF-2α). Targeting this survival mechanism has longly been attempted with mixed results, in many tumors. An interesting success was recently obtained in Renal Cell Carcinoma associated with the Von Hippel-Lindau disease, by the HIF-2α inhibitor Belzutifan, which reached an ORR of 49% (Jonasch et al., 2021).

In MMe, this approach has been exploited only recently and in a preclinical setting. Shukuya et al., for example, demonstrated that MMe cells that carry a mutation of the metabolic regulator VHL (Von Hippel Lindau) can sensibly slow down their growth rate when exposed to the HIF-1α inhibitor YC-1 (Shukuya et al., 2020). Similarly, Li Petri et al. showed that MMe cells tend to seek a hypoxic status in order to decrease the expression of proton-coupled folate transporter (PCFT), which is associated with improved survival of MMe patients treated with pemetrexed (Li Petri et al., 2020). In the same work, they demonstrated, both *in vitro* and *in vivo,* that the inhibition of the lactate dehydrogenase enzyme through the novel inhibitor NHI-Glc-2 causes the disruption of the MMe spheroid cell cultures and exert a marked antitumor efficacy in the mice. The effect of this agent seems to be synergistic with some of the chemotherapy drugs already used in the clinical practice, such as Pemetrexed and Gemcitabine.

## Conclusion

Until recently, MMe was an orphan disease, endowed by a dramatic prognosis which benefited little from available, mainly chemotherapy-based, treatments.

More recently, ICIs have represented an improvement in the palliative treatment setting of this disease; although, this improvement proved to be small, it represents a first step towards more active novel treatment options.

Unbiased trials, possibly conducted with novel designs (e.g., adaptive studies) are still needed, also in order to improve the cost:ratio benefit of the next generation of agents. Indeed, the small amount of benefit achieved so far should be weighted against the costs of novel anticancer agents and strategies.

The preclinical and early clinical studies here discussed offer a glimpse of what the therapy of MMe could look like in the next future, offering hopes for our patients. It is however clear, in our opinion, that the road towards more effective treatments for MMe ahead of us is still long and complex.
